# Autopsy Pathology’s Paradigm Shift: Artificial Intelligence and Emerging Technologies in the Era of Digitally Integrated Death Investigation

**DOI:** 10.3390/diagnostics16152405

**Published:** 2026-07-30

**Authors:** Ivan Dieb Miziara, Carmen Silvia Molleis Galego Miziara

**Affiliations:** Department of Legal Medicine, Bioethics, Occupational Medicine and Physical Medicine and Rehabilitation, Faculdade de Medicina da Universidade de São Paulo, São Paulo 05405-000, Brazil

**Keywords:** artificial intelligence, forensic pathology, autopsy, virtopsy, postmortem imaging, digital pathology, computational pathology, molecular autopsy, precision forensic medicine, forensic radiology

## Abstract

**Background:** Autopsy pathology remains the reference standard for determining the cause of death, reconstructing disease and injury mechanisms, ensuring diagnostic quality, and supporting medical education and forensic investigations. However, declining autopsy rates, workforce shortages, biosafety concerns, increasing diagnostic complexity, and the rapid evolution of digital technologies have stimulated the development of complementary investigative approaches. This review critically examines whether artificial intelligence (AI) and emerging technologies are driving a genuine paradigm shift toward digitally integrated death investigation. **Methods:** A structured narrative review informed by a systematic literature search was conducted in PubMed/MEDLINE, Embase, Scopus, and Web of Science, covering publications from January 2000 through June 2026. Evidence addressing postmortem imaging, virtopsy, digital pathology, computational pathology, molecular autopsy, robotics, artificial intelligence, machine learning, and emerging omics technologies was critically appraised. Owing to the methodological heterogeneity of the available literature, findings were synthesized qualitatively according to technological maturity, forensic applicability, validation status, and implementation readiness. **Results:** The reviewed evidence demonstrates substantial progress in postmortem computed tomography, postmortem CT angiography, postmortem magnetic resonance imaging, whole-slide imaging, molecular autopsy, robotic-assisted postmortem procedures, three-dimensional reconstruction, and AI-assisted forensic analysis. These technologies enhance trauma evaluation, vascular imaging, ballistic reconstruction, digital documentation, remote consultation, diagnostic reproducibility, and multimodal integration of forensic evidence. Nevertheless, the level of evidence varies considerably across technological domains. Postmortem imaging represents the most mature and extensively validated technology, whereas most AI applications remain supported predominantly by retrospective proof-of-concept studies with limited multicenter external validation. Current systematic evidence further indicates that AI should presently be regarded as an assistive technology that augments expert forensic interpretation rather than replacing conventional autopsy or autonomous medicolegal decision-making. **Conclusions:** Contemporary autopsy pathology is evolving toward a hybrid model of digitally integrated death investigation in which conventional autopsy, imaging, digital pathology, molecular diagnostics, robotics, and AI function as complementary components of a unified forensic workflow. Current evidence supports a conceptual paradigm shift characterized by transformation of evidence acquisition, preservation, interpretation, and integration, while reaffirming that conventional autopsy remains the indispensable biological reference standard for the development, validation, and medicolegal interpretation of all emerging technologies. Future implementation should prioritize multicenter validation, standardized forensic datasets, explainable AI, digital chain-of-custody procedures, and robust regulatory governance to ensure safe and scientifically reliable integration into forensic practice.

## 1. Introduction

For more than two centuries, autopsy pathology has represented the reference standard for investigating disease, establishing the cause of death, validating clinical diagnoses, and advancing medical knowledge. Beginning with the clinicopathological correlations established by Morgagni and subsequently refined through Virchow’s concept of cellular pathology, the autopsy evolved into one of the most influential investigative tools in medicine, providing the biological foundation for diagnostic quality assurance, medical education, epidemiological surveillance, and medicolegal investigation [1,2,3,4]. Despite remarkable advances in clinical imaging, laboratory diagnostics, and molecular medicine, numerous studies continue to demonstrate that conventional autopsy remains the most reliable method for identifying major diagnostic discrepancies and accurately determining the cause of death, reaffirming its role as the biological reference standard against which other diagnostic modalities are validated [2,3,4,5].

Paradoxically, the scientific relevance of autopsy has persisted despite a sustained worldwide decline in hospital and forensic autopsy rates. Financial constraints, shortages of trained pathologists, increasing workload, biosafety concerns, cultural and religious considerations, and growing confidence in sophisticated antemortem diagnostic technologies have collectively reduced the frequency of conventional autopsies in many healthcare systems [5,6,7,8,9]. The COVID-19 pandemic temporarily reversed this trend by demonstrating the irreplaceable value of postmortem examination in characterizing diffuse alveolar damage, endothelial injury, microvascular thrombosis, and multisystem organ involvement before these pathological mechanisms were fully recognized clinically [6,7]. These observations reinforced an important concept: rather than becoming obsolete, autopsy continues to generate the pathological ground truth upon which emerging diagnostic technologies depend.

The response to these challenges has not been the replacement of conventional autopsy but the progressive incorporation of complementary digital technologies. Among these, postmortem imaging—including postmortem computed tomography (PMCT), postmortem magnetic resonance imaging (PMMRI), postmortem computed tomography angiography (PMCTA), and three-dimensional reconstruction—has become the most mature component of contemporary forensic investigation. Since the pioneering Virtopsy Project introduced by Dirnhofer, Thali, and colleagues, these techniques have demonstrated substantial value in trauma assessment, ballistic reconstruction, vascular injury evaluation, disaster victim identification, minimally invasive postmortem investigation, and permanent digital documentation while preserving anatomical integrity [10,11,12,13,14,15,16]. Extensive validation studies consistently indicate that postmortem imaging complements rather than replaces conventional autopsy, particularly because many natural diseases, inflammatory disorders, toxicological deaths, and microscopic pathological processes remain dependent on direct tissue examination.

Digital pathology represents a second major pillar of this technological transformation. Whole-slide imaging (WSI), computational pathology, quantitative morphometry, and artificial intelligence (AI)-assisted image analysis have profoundly changed diagnostic pathology by enabling permanent digital archiving, remote consultation, computational analysis, and improved reproducibility [17,18,19,20,21,22,23]. However, forensic implementation remains considerably less mature than clinical pathology because autopsy tissues present unique biological characteristics, including autolysis, decomposition, variable fixation, thermal injury, and heterogeneous staining patterns that challenge algorithms developed using surgical pathology specimens. Recent systematic reviews emphasize that forensic digital pathology remains an emerging discipline requiring standardized validation protocols, interoperable digital workflows, quality assurance procedures, and secure digital chain-of-custody standards before routine implementation can be recommended [22,23].

Artificial intelligence has emerged as one of the most rapidly expanding fields within forensic medicine. Machine learning and deep learning algorithms have demonstrated promising performance in fracture detection, intracranial hemorrhage recognition, firearm wound classification, pulmonary fat embolism detection, automated diatom identification, quantitative histopathology, anthropological analysis, and postmortem interval estimation [24,25,26,27,28,29,30,31,32]. Nevertheless, recent systematic reviews dedicated specifically to AI in forensic pathology consistently conclude that the current evidence remains dominated by retrospective proof-of-concept studies, relatively small single-center datasets, heterogeneous validation strategies, and limited multicenter external validation [31,32]. Consequently, AI should currently be regarded as a decision-support technology that augments expert forensic interpretation rather than replacing conventional autopsy or autonomous medicolegal decision-making.

At the same time, molecular autopsy and emerging omics technologies have expanded the diagnostic scope of forensic pathology beyond conventional morphology. Postmortem genetic testing has become an established adjunct in the investigation of sudden unexplained death associated with inherited arrhythmogenic disorders and cardiomyopathies, while transcriptomics, proteomics, metabolomics, epigenetics, microbiome analysis, and microRNA profiling are opening new perspectives for disease characterization, wound vitality assessment, and postmortem interval estimation [33,34,35,36,37,38]. Although many of these technologies remain investigational, together they illustrate the progressive transition from morphology-based diagnosis toward multidimensional forensic investigation.

Robotic-assisted postmortem systems, image-guided biopsy, three-dimensional digital reconstruction, computational pathology, and multimodal data integration further illustrate that contemporary forensic pathology is no longer defined by isolated technological innovations. Instead, modern death investigation increasingly relies on the interaction of radiological, pathological, molecular, toxicological, computational, and digital sources of evidence that collectively support more comprehensive forensic interpretation [15,39]. Simultaneously, the rapid expansion of AI has raised important ethical, regulatory, and legal challenges involving algorithmic bias, explainability, transparency, cybersecurity, digital chain of custody, and judicial admissibility. International initiatives—including the World Health Organization guidance on AI for health, the European Union Artificial Intelligence Act, and recent United States Food and Drug Administration recommendations—highlight that robust governance and continuous validation are indispensable prerequisites for implementing AI in high-risk domains such as forensic medicine [40,41,42,43,44,45].

Although numerous reviews have examined individual technologies—including virtopsy, postmortem imaging, digital pathology, molecular autopsy, or artificial intelligence—few have critically evaluated these developments as components of a single, evolving forensic ecosystem. Existing reviews frequently emphasize technological innovation while providing comparatively limited analysis of technological maturity, validation quality, forensic applicability, implementation readiness, and the continuing dependence of emerging technologies on conventional autopsy. Moreover, the most recent systematic reviews indicate that the scientific evidence supporting many AI applications remains substantially less mature than commonly perceived, reinforcing the need for a balanced and evidence-based appraisal of their current role within forensic practice [31,32,46].

Accordingly, the present study was designed as a structured narrative review informed by a systematic literature search to critically evaluate the current evidence regarding artificial intelligence and emerging technologies in autopsy pathology. Rather than asking whether digital technologies will replace conventional autopsy, this review examines whether their progressive convergence supports the emergence of a hybrid, digitally integrated model of death investigation. Particular emphasis is placed on technological maturity, methodological robustness, forensic validation, ethical governance, regulatory implications, and the continuing role of conventional autopsy as the indispensable biological reference standard upon which all emerging technologies continue to depend.

## 2. Materials and Methods

### 2.1. Study Design

This study was conducted as a structured narrative review informed by a systematic literature search to critically evaluate the current evidence regarding artificial intelligence (AI) and emerging technologies in autopsy pathology and forensic medicine. The primary objective was not to estimate pooled effect sizes or perform quantitative comparisons but to synthesize heterogeneous evidence originating from multiple technological domains, evaluate the maturity of the available evidence, identify methodological limitations, and assess the implications of these technologies for digitally integrated death investigation.

A structured narrative approach was selected because the available literature encompasses diverse study designs—including prospective validation studies, retrospective observational investigations, technical feasibility studies, proof-of-concept computational models, systematic reviews, narrative reviews, methodological reports, consensus statements, and regulatory documents—precluding meaningful quantitative meta-analysis while favoring critical qualitative integration.

To maximize methodological transparency, the literature identification and selection process followed the principles of the Preferred Reporting Items for Systematic Reviews and Meta-Analyses (PRISMA 2020) whenever applicable to structured narrative reviews.

### 2.2. Information Sources

A comprehensive literature search was performed using four major biomedical databases:

- PubMed/MEDLINE
- Embase
- Scopus
- Web of Science

The search covered publications from 1 January 2000 through 20 June 2026.

The year 2000 was predefined because it marks the beginning of the modern digital transformation of autopsy pathology, including the emergence of multislice postmortem CT, the Virtopsy Project, molecular autopsy, digital pathology, computational pathology, and later artificial intelligence applied to forensic medicine.

Reference lists of all eligible publications, landmark studies, systematic reviews, consensus statements, and international guidance documents were manually screened to identify additional influential publications not retrieved electronically.

Only publications written in English, Portuguese, or Spanish were considered.

### 2.3. Literature Search Strategy

Search strategies were adapted to the indexing system of each database using controlled vocabulary (MeSH and Emtree) combined with free-text terms.

The principal search domains included:

- artificial intelligence;
- machine learning;
- deep learning;
- computational pathology;
- digital pathology;
- whole-slide imaging;
- forensic pathology;
- autopsy;
- virtopsy;
- postmortem imaging;
- PMCT;
- PMMRI;
- PMCTA;
- molecular autopsy;
- robotics;
- image-guided biopsy;
- precision forensic medicine.

A representative PubMed strategy combined these concepts using Boolean operators. Equivalent strategies for Embase, Scopus, and Web of Science are provided in Supplementary Material File S1.

### 2.4. Eligibility Criteria

Studies were considered eligible when they:

1. addressed autopsy pathology, forensic pathology, forensic radiology, postmortem investigation, or digitally integrated death investigation;
2. evaluated at least one emerging technology relevant to forensic practice;
3. were published in peer-reviewed journals or official documents issued by recognized international organizations;
4. provided sufficient methodological information for critical appraisal.

Eligible study designs included:

- prospective validation studies;
- retrospective observational investigations;
- diagnostic accuracy studies;
- technical feasibility studies;
- methodological investigations;
- proof-of-concept studies;
- systematic reviews;
- high-quality narrative reviews;
- consensus statements;
- professional guidelines.

Publications were excluded if they:

- focused exclusively on clinical applications without forensic relevance;
- were conference abstracts without full methodology;
- consisted solely of editorials, letters, or opinion articles;
- represented duplicate publications;
- were non-peer-reviewed preprints;
- lacked sufficient bibliographic information.

### 2.5. Study Selection

After duplicate removal, titles and abstracts were screened independently by both authors.

Potentially eligible studies underwent full-text assessment.

Disagreements regarding eligibility were resolved through discussion and consensus.

**Figure 1:**
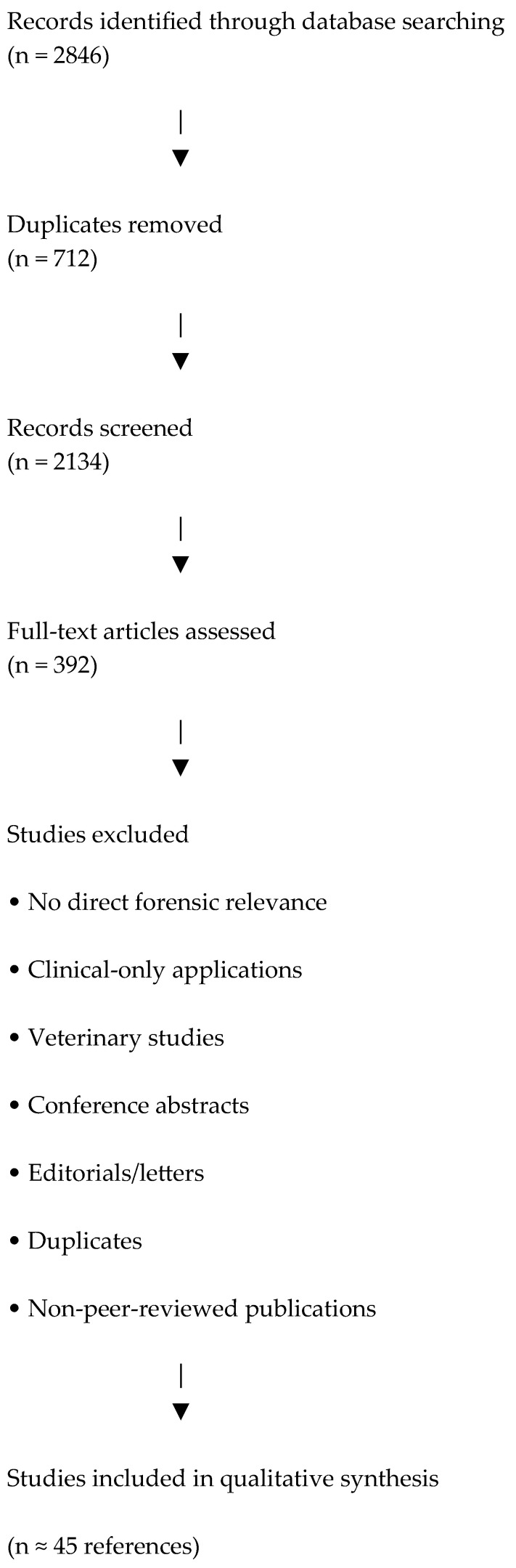
PRISMA flow diagram summarizing the identification, screening, eligibility assessment, and inclusion of studies in this structured narrative review.

The complete identification and selection process is summarized in the PRISMA flow diagram (Figure 1).

### 2.6. Data Extraction

Data extraction followed a predefined Evidence Extraction Sheet developed specifically for this review.

For each study, the following information was collected:

- authors;
- publication year;
- country;
- technological domain;
- study design;
- population or dataset;
- forensic application;
- comparator (when applicable);
- principal outcomes;
- diagnostic performance;
- validation status;
- methodological limitations;
- applicability to forensic practice.

### 2.7. Evidence Classification

Because technologies differed substantially in maturity and methodological design, studies were classified according to their principal scientific contribution:

- landmark studies;
- primary validation studies;
- technical feasibility studies;
- systematic reviews;
- high-quality narrative reviews;
- consensus statements;
- international regulatory documents.

This framework allowed interpretation based on evidentiary strength rather than publication type alone.

### 2.8. Technology Maturity Assessment

Technological maturity was evaluated qualitatively using five predefined categories:

- Established
- Advanced validation
- Early validation
- Proof of concept
- Experimental

Classification considered:

- forensic implementation;
- external validation;
- multicenter evidence;
- reproducibility;
- routine clinical or forensic use.

### 2.9. Critical Appraisal

Because the review incorporated heterogeneous study designs, a single formal risk-of-bias instrument was not considered appropriate.

Instead, all included studies underwent structured qualitative appraisal according to four methodological domains:

- methodological robustness;
- forensic applicability;
- validation status;
- implementation limitations.

Particular attention was paid to distinguishing evidence derived directly from forensic material from studies extrapolated from clinical medicine.

### 2.10. Narrative Synthesis

Evidence was synthesized according to six predefined technological domains:

1. postmortem imaging and virtopsy;
2. AI-assisted postmortem imaging;
3. digital pathology;
4. molecular autopsy and omics;
5. robotics and minimally invasive autopsy;
6. governance, regulation, and implementation.

Within each domain, emphasis was placed on:

- technological advances;
- landmark validation studies;
- diagnostic performance;
- implementation barriers;
- remaining knowledge gaps.

### 2.11. Artificial Intelligence-Specific Methodological Considerations

Because AI studies are particularly susceptible to methodological bias, additional variables were considered during critical appraisal, including:

- dataset origin;
- annotation quality;
- sample size;
- class balance;
- forensic versus clinical datasets;
- internal versus external validation;
- explainability;
- generalizability.

Algorithms developed exclusively using clinical material were not considered directly validated for forensic implementation unless subsequent postmortem validation had been reported.

### 2.12. Reporting Standards

The manuscript was prepared according to the PRISMA 2020 reporting principles whenever applicable to structured narrative reviews.

Given the conceptual objective of the review and the marked heterogeneity of the available evidence, quantitative meta-analysis, formal certainty-of-evidence grading, and statistical heterogeneity assessment were intentionally not performed.

Methodological transparency was ensured through explicit eligibility criteria, standardized evidence extraction, structured evidence classification, qualitative critical appraisal, technology maturity assessment, and transparent narrative synthesis.

### 2.13. Methodological Workflow

The methodological pipeline comprised:

1. systematic literature search;
2. duplicate removal;
3. title and abstract screening;
4. full-text eligibility assessment;
5. standardized data extraction;
6. evidence classification;
7. technology maturity assessment;
8. qualitative critical appraisal;
9. thematic narrative synthesis;
10. integrated interpretation focused on forensic applicability, validation status, and implementation readiness.

This methodological framework was designed to provide a balanced and evidence-based assessment of emerging technologies while acknowledging the heterogeneity of the available literature and the rapidly evolving nature of digitally integrated forensic pathology.

## 3. Results

### 3.1. Literature Search and Characteristics of the Included Evidence

The literature identification process is summarized in Figure 1. Database searching yielded 2846 records, of which 712 duplicates were removed. Following title and abstract screening, 392 publications underwent full-text assessment. After application of the predefined eligibility criteria, the final corpus comprised the landmark studies, systematic reviews, validation investigations, methodological reports, and international guidance documents considered most relevant for evaluating artificial intelligence and emerging technologies in autopsy pathology. The landmark studies are summarized in Table 1.

The included publications demonstrated marked methodological heterogeneity, encompassing prospective validation studies, retrospective observational investigations, diagnostic accuracy studies, technical feasibility reports, proof-of-concept AI studies, systematic reviews, narrative reviews, consensus statements, and regulatory documents.

The evidence naturally clustered into six major technological domains:

1. Postmortem imaging and Virtopsy.
2. Artificial intelligence-assisted postmortem imaging.
3. Digital pathology and computational pathology.
4. Molecular autopsy and emerging omics.
5. Robotic-assisted postmortem investigation.
6. Governance, validation, and regulatory implementation.

Rather than representing technologies at equivalent stages of development, these domains demonstrated substantial differences in methodological robustness, validation status, and forensic implementation.

### 3.2. Technology Maturity Across Forensic Domains

The literature consistently demonstrated that technological maturity varies considerably among emerging forensic technologies.

Postmortem imaging, particularly PMCT and PMCTA, represents the most mature technological domain, supported by prospective validation studies, multicenter implementation, and more than two decades of accumulated forensic experience. These modalities have become established adjuncts in trauma investigation, ballistic reconstruction, vascular injury assessment, disaster victim identification, and minimally invasive autopsy.

Molecular autopsy occupies an intermediate position. Although applicable only to selected diagnostic scenarios, particularly sudden unexplained cardiac death, it is supported by robust clinical evidence and has become an established complementary investigation in specialized centers.

Digital pathology has reached widespread implementation in clinical pathology but remains in an earlier phase within forensic medicine. Current evidence supports high diagnostic concordance between whole-slide imaging and conventional microscopy, although routine forensic implementation remains limited.

Artificial intelligence demonstrated the greatest discrepancy between scientific enthusiasm and evidentiary maturity. Although numerous applications have reported encouraging diagnostic performance, most studies remain retrospective, single-center investigations with limited external validation.

Robotic-assisted autopsy currently represents the least mature technological domain, with evidence largely restricted to technical feasibility studies.

A comparative overview of technological maturity is presented in Table 2.

### 3.3. Postmortem Imaging and Virtopsy

Postmortem imaging remains the most extensively validated technology identified in the present review.

Across multiple prospective investigations, PMCT consistently demonstrated excellent performance for:

- skeletal trauma;
- firearm injuries;
- intracranial hemorrhage;
- gas embolism;
- foreign-body localization;
- ballistic reconstruction.

The subsequent development of PMCTA substantially improved vascular visualization, allowing more accurate investigation of arterial disruption, aneurysms, traumatic hemorrhage, and cardiovascular injury.

Recent systematic reviews consistently conclude that postmortem imaging has evolved from an experimental adjunct into an established component of modern forensic investigation. Nevertheless, imaging remains insufficient for several pathological conditions—including myocarditis, early myocardial infarction, sepsis, metabolic disorders, intoxication, and microscopic inflammatory diseases—that continue to require conventional histopathological examination.

Collectively, the available evidence indicates that imaging should be interpreted as a complementary modality rather than a substitute for conventional autopsy.

### 3.4. Artificial Intelligence in Forensic Pathology

Artificial intelligence represents one of the fastest-growing areas of forensic research.

The reviewed literature identified applications involving:

- PMCT interpretation;
- intracranial hemorrhage detection;
- fracture recognition;
- firearm wound classification;
- pulmonary fat embolism;
- diatom identification;
- quantitative histopathology;
- anthropological image analysis.

Most reported algorithms achieved high internal diagnostic performance.

However, recent systematic reviews consistently identified common methodological limitations:

- retrospective study design;
- relatively small datasets;
- single-center development;
- heterogeneous validation protocols;
- scarcity of prospective multicenter validation.

Importantly, many forensic AI algorithms were originally adapted from clinical imaging or surgical pathology rather than developed using authentic postmortem material.

Consequently, the present evidence supports AI primarily as a decision-support technology that augments expert forensic interpretation rather than replacing conventional medicolegal reasoning.

### 3.5. Digital Pathology and Computational Pathology

Digital pathology has undergone rapid technological development.

Whole-slide imaging enables permanent digital archiving, remote consultation, computational analysis, quantitative morphometry, and integration with AI algorithms.

Validation studies demonstrated excellent diagnostic concordance between digital slides and conventional microscopy under controlled forensic conditions.

Nevertheless, forensic implementation remains less mature than clinical pathology because postmortem tissues frequently exhibit:

- autolysis;
- decomposition;
- fixation variability;
- thermal injury;
- heterogeneous staining.

Recent systematic reviews concluded that standardized validation protocols, interoperability standards, quality assurance procedures, and digital chain-of-custody frameworks remain prerequisites for routine implementation.

### 3.6. Molecular Autopsy and Emerging Omics

Molecular autopsy has become an established adjunct for investigating sudden unexplained death associated with inherited arrhythmogenic disorders.

Beyond targeted genetic testing, emerging technologies—including transcriptomics, proteomics, metabolomics, epigenetics, microbiome analysis, and microRNA profiling—demonstrated promising applications in:

- disease characterization;
- wound vitality assessment;
- postmortem interval estimation.

However, these technologies remain largely investigational owing to methodological heterogeneity, limited validation cohorts, tissue degradation, and the absence of standardized analytical pipelines.

### 3.7. Robotics and Image-Guided Postmortem Investigation

Robotic-assisted postmortem systems remain supported primarily by feasibility studies.

Current applications include:

- robotic biopsy;
- automated three-dimensional body scanning;
- navigated tissue sampling;
- digital reconstruction.

Although these technologies improve standardization and documentation, no convincing evidence currently supports replacement of conventional autopsy.

Their principal contribution lies in image-guided minimally invasive investigation integrated with PMCT.

### 3.8. Validation, Governance, and Implementation Readiness

Across all technological domains, one finding emerged consistently.

Scientific innovation has progressed more rapidly than forensic validation.

The strongest evidence currently supports PMCT, PMCTA, and molecular autopsy.

Digital pathology has entered early forensic implementation.

Artificial intelligence remains predominantly supported by retrospective proof-of-concept investigations.

Robotic systems remain experimental.

Recent regulatory initiatives issued by the WHO, the European Union, and the FDA consistently emphasize that future implementation depends not only on diagnostic performance but also on external validation, explainability, quality assurance, digital chain of custody, cybersecurity, and human oversight.

### 3.9. Integrated Interpretation of the Current Evidence

Taken together, the reviewed literature demonstrates that contemporary forensic pathology is undergoing progressive technological convergence rather than isolated innovation.

Postmortem imaging, digital pathology, molecular diagnostics, computational pathology, robotics, and artificial intelligence increasingly function as complementary components of a unified investigative workflow.

At the same time, all emerging technologies continue to depend on conventional autopsy for biological validation, pathological correlation, and medicolegal interpretation.

Accordingly, the available evidence supports the emergence of a hybrid model of digitally integrated death investigation in which conventional autopsy remains the indispensable biological reference standard while digital technologies progressively expand diagnostic capability, reproducibility, documentation, and multidisciplinary integration.

## 4. Discussion

### 4.1. Principal Findings

The present review provides a comprehensive critical synthesis of the current evidence regarding artificial intelligence (AI) and emerging technologies in autopsy pathology. Unlike previous reviews that have generally examined individual technological domains in isolation, this study integrated evidence from postmortem imaging, digital pathology, computational pathology, molecular autopsy, robotics, artificial intelligence, and emerging regulatory frameworks within a unified conceptual perspective. By critically evaluating technological maturity, methodological robustness, forensic validation, institutional implementation, and medicolegal implications, the review sought not merely to summarize recent advances but to determine whether the available evidence supports a broader transformation of contemporary forensic pathology.

The first principal finding is that the current technological transformation should not be understood as the progressive replacement of conventional autopsy. Rather, the reviewed literature consistently demonstrates the emergence of increasingly integrated investigative workflows in which conventional autopsy, postmortem imaging, digital pathology, molecular diagnostics, computational analysis, and artificial intelligence operate as complementary components of a unified diagnostic process. Throughout this review, this transformation has been described as digitally integrated death investigation, emphasizing that technological innovation derives its greatest value from multimodal integration rather than from the isolated performance of individual technologies.

The second major finding concerns the current role of artificial intelligence. Although AI has become one of the fastest-growing research areas in forensic medicine and has demonstrated encouraging performance across numerous narrowly defined analytical tasks, the available evidence consistently indicates that most published applications remain supported by retrospective proof-of-concept studies, relatively small single-center datasets, and limited external validation [31,32,46]. Recent systematic reviews therefore converge on the conclusion that AI should presently be regarded as a decision-support technology that augments expert forensic interpretation rather than replacing conventional medicolegal reasoning. This concept of augmented forensic intelligence provides a more balanced and scientifically defensible interpretation of the current state of forensic AI than narratives emphasizing imminent autonomous forensic decision-making.

A third important observation is that the reviewed evidence collectively supports the emergence of Precision Forensic Pathology. Rather than representing the simple transfer of concepts from precision medicine into forensic practice, this framework reflects the progressive integration of anatomical examination, postmortem imaging, digital pathology, molecular diagnostics, toxicology, computational analysis, and contextual medicolegal information into a multidimensional diagnostic model. Current evidence suggests that forensic pathology is progressively moving beyond an exclusively morphology-based discipline toward a multimodal interpretative framework while maintaining conventional autopsy as its biological foundation.

Importantly, the evidence synthesized throughout this review also demonstrates that technological innovation alone is insufficient for responsible forensic implementation. Recent systematic reviews, validation studies, and international regulatory initiatives consistently emphasize the need for robust scientific validation, external multicenter evaluation, explainability, digital governance, cybersecurity, quality assurance, and human oversight before emerging technologies can be incorporated into routine medicolegal practice [21,22,23,31,32,40,41,42,43,44,45,46]. Accordingly, the conceptual Forensic Validation Framework and Institutional Readiness Framework proposed in this review synthesize these converging recommendations into complementary models intended to guide the responsible implementation of digitally integrated forensic technologies.

When these observations are interpreted collectively, a broader conceptual conclusion emerges. The reviewed literature demonstrates simultaneous transformations in the acquisition, preservation, interpretation, validation, governance, and institutional implementation of forensic evidence. Considered individually, each technological advance may be interpreted as an incremental innovation. Considered collectively, however, these interconnected developments fundamentally modify the conceptual framework through which medicolegal investigation is conducted. Interpreted within the epistemological perspective proposed by Thomas Kuhn [47], these changes are consistent with the characteristics of a genuine scientific paradigm shift.

Importantly, the paradigm shift identified in the present review should not be interpreted as technological substitution. On the contrary, every major technological domain reviewed continues to depend upon conventional autopsy for biological validation, clinicopathological correlation, algorithm development, and medicolegal interpretation. Conventional autopsy therefore retains a unique epistemological position within contemporary forensic medicine, functioning as the biological reference standard upon which digitally integrated death investigation continues to depend.

Collectively, the findings synthesized throughout this review suggest that the future of forensic pathology will be determined not by the success of any individual technology but by the successful integration of validated imaging, pathology, molecular diagnostics, computational analysis, artificial intelligence, quality assurance, and institutional governance within scientifically robust and legally reliable investigative systems. The transformation currently underway therefore represents not the decline of conventional autopsy but its evolution into the central biological component of an increasingly integrated, computationally augmented, and digitally enabled model of death investigation.

### 4.2. From Conventional Autopsy to Digitally Integrated Death Investigation

The discussion is organized as a progressive interpretation of the evidence synthesized in the Results. For more than two centuries, conventional autopsy has constituted the cornerstone of pathological diagnosis and forensic medicine. Since the clinicopathological correlations established by Morgagni and the subsequent development of cellular pathology by Virchow, postmortem examination has served as the definitive method for correlating clinical manifestations with structural disease, validating premortem diagnoses, determining the cause of death, and supporting medical education, epidemiological surveillance, and medicolegal investigation [1,2,3,4,5]. Despite remarkable advances in clinical imaging, laboratory diagnostics, and precision medicine, large observational studies and systematic reviews continue to demonstrate that conventional autopsy remains the biological reference standard for identifying major diagnostic discrepancies and establishing definitive clinicopathological correlations [2,3,4,5].

Paradoxically, the continued scientific importance of autopsy has coincided with a sustained worldwide decline in hospital autopsy rates. Multiple studies have attributed this reduction to financial constraints, shortages of trained pathologists, increasing clinical workload, cultural and religious concerns, biosafety issues, and growing confidence in increasingly sophisticated antemortem diagnostic technologies [5,8,9]. Rather than diminishing the scientific importance of autopsy, however, these challenges have stimulated the development of complementary technologies capable of improving documentation, reproducibility, accessibility, and diagnostic efficiency while preserving the evidentiary reliability of conventional pathological examination.

The most mature response to these challenges has been the development of postmortem imaging. During the past two decades, PMCT, PMMRI, PMCTA, three-dimensional surface scanning, and digital reconstruction have progressively evolved from experimental techniques into established components of forensic investigation [10,11,12,13,14,15,16]. Landmark studies conducted by Thali, Dirnhofer, Roberts, Grabherr, and colleagues consistently demonstrated that postmortem imaging provides major advantages in skeletal trauma, ballistic reconstruction, vascular injury assessment, foreign-body localization, gas embolism detection, disaster victim identification, and minimally invasive postmortem investigation [10,11,12,13,14,15,16]. At the same time, prospective validation studies have repeatedly shown that imaging alone remains insufficient for diagnosing many natural diseases—including myocarditis, early myocardial infarction, inflammatory disorders, metabolic diseases, intoxications, and numerous microscopic pathological processes—that continue to require conventional histopathological examination [13,14,15,16]. Consequently, the current literature consistently supports postmortem imaging as a complementary extension of conventional autopsy rather than as its replacement.

A comparable evolution has occurred in digital pathology. The introduction of whole-slide imaging and computational pathology has fundamentally changed histopathological practice by enabling permanent digital archiving, remote consultation, quantitative image analysis, and computational interpretation [17,18,19,20,21,22,23]. Large validation studies performed in clinical pathology demonstrated that digital pathology can achieve diagnostic performance comparable with conventional microscopy under appropriate quality-control conditions [17,18,19,20]. More recently, forensic-specific investigations have confirmed high diagnostic concordance between whole-slide imaging and conventional histological examination while simultaneously emphasizing that forensic implementation presents unique challenges related to autolysis, decomposition, fixation variability, postmortem artifacts, and heterogeneous tissue preservation [21,22,23]. Recent systematic reviews therefore conclude that standardized validation protocols, interoperability standards, quality assurance procedures, and secure digital chain-of-custody frameworks remain essential prerequisites for widespread implementation of digital pathology in forensic medicine [22,23].

Molecular autopsy has expanded forensic investigation beyond conventional structural pathology by introducing a complementary molecular dimension to cause-of-death determination. Robust evidence now supports postmortem genetic testing in selected cases of sudden unexplained death associated with inherited arrhythmogenic disorders and cardiomyopathies [33,34]. Simultaneously, emerging omics technologies—including transcriptomics, epigenetics, proteomics, metabolomics, microbiome analysis, and microRNA profiling—have demonstrated promising applications in disease characterization, wound vitality assessment, and estimation of the postmortem interval, although current evidence remains largely exploratory because of methodological heterogeneity, limited validation cohorts, and the absence of standardized analytical pipelines [35,36,37,38]. Collectively, these developments indicate that contemporary forensic diagnosis increasingly integrates structural pathology with molecular biology rather than relying exclusively on anatomical findings.

Artificial intelligence represents the most recent layer within this technological evolution. Recent systematic reviews demonstrate rapid expansion of AI applications across postmortem imaging, digital pathology, forensic anthropology, wound interpretation, histopathological quantification, and multimodal data analysis [31,32,46]. Nevertheless, these same reviews consistently conclude that the overwhelming majority of published studies remain retrospective, based on relatively small single-center datasets, and supported by limited multicenter external validation [31,32,46]. Importantly, many currently available algorithms were originally developed using clinical imaging or surgical pathology material before being adapted to forensic applications. Consequently, the literature increasingly supports AI as a decision-support technology capable of augmenting expert interpretation while emphasizing that autonomous medicolegal decision-making remains unsupported by current evidence [24,25,26,27,28,29,30,31,32,46].

Beyond the individual performance of each technology, a broader pattern emerges from the available evidence. Recent publications no longer describe postmortem imaging, digital pathology, molecular autopsy, computational pathology, or artificial intelligence as isolated innovations. Instead, they increasingly portray these technologies as interconnected components of multidisciplinary forensic workflows in which radiological, anatomical, histopathological, molecular, toxicological, and computational information are interpreted collectively to improve diagnostic accuracy and reproducibility [15,21,22,23,27,31,32]. International guidance issued by the World Health Organization, the European Union, and the United States Food and Drug Administration further reinforces this perspective by emphasizing that implementation of AI-enabled technologies should occur within integrated governance frameworks based on transparency, explainability, continuous validation, human oversight, and robust quality assurance [42,43,44,45].

Taken together, these observations support an important conceptual interpretation. The principal transformation currently occurring in forensic pathology is not the progressive replacement of conventional autopsy by digital technologies. Rather, the literature demonstrates the emergence of increasingly integrated investigative workflows in which conventional autopsy remains the biological reference standard while imaging, digital pathology, molecular diagnostics, computational pathology, robotics, and artificial intelligence expand its diagnostic capabilities. Contemporary forensic investigation therefore appears to be evolving toward a digitally integrated model of death investigation, characterized by multimodal acquisition, preservation, interpretation, and communication of forensic evidence. Importantly, this concept does not imply technological substitution. On the contrary, every major technological domain reviewed continues to depend on conventional autopsy for biological validation, pathological correlation, algorithm development, and medicolegal interpretation. The available evidence therefore supports integration rather than replacement as the defining characteristic of contemporary forensic pathology.

### 4.3. Artificial Intelligence as Augmented Forensic Intelligence

Artificial intelligence has become one of the fastest-growing areas of research in forensic medicine during the past decade. Machine learning and deep learning algorithms have been applied to a wide range of forensic tasks, including postmortem imaging, wound classification, fracture detection, intracranial hemorrhage recognition, forensic anthropology, histopathological image analysis, pulmonary fat embolism detection, automated diatom identification, estimation of the postmortem interval, and multimodal forensic data analysis [24,25,26,27,28,29,30,31,32]. Collectively, these studies demonstrate that AI has considerable potential to improve computational image analysis, automate repetitive analytical tasks, reduce observer variability, and facilitate the quantitative interpretation of increasingly complex forensic datasets.

The strongest evidence currently available originates from highly specific diagnostic applications rather than comprehensive forensic case interpretation. Several primary validation studies have reported encouraging diagnostic performance for narrowly defined analytical tasks. Deep-learning algorithms have demonstrated high accuracy for detecting intracranial hemorrhage on postmortem computed tomography, identifying skeletal fractures, classifying firearm wounds, recognizing pulmonary fat embolism in digitized histopathological slides, and automating diatom identification during drowning investigations [28,29,30]. Similar advances have been reported in computational pathology, where machine-learning algorithms have shown promising performance in tissue classification, quantitative morphometry, lesion segmentation, and image-based biomarker prediction [17,18,19,20,21]. These studies collectively indicate that AI is capable of performing selected analytical tasks with high reproducibility under controlled experimental conditions.

However, systematic evaluation of the available evidence reveals important methodological limitations. Three recent systematic reviews dedicated to AI in forensic sciences reached remarkably consistent conclusions despite evaluating different technological domains [31,32,46]. Across these reviews, most published studies were retrospective, conducted within single institutions, based on relatively small datasets, and focused on proof-of-concept algorithm development rather than prospective clinical implementation. External multicenter validation remained uncommon, standardized benchmarking datasets were scarce, and direct comparisons between competing algorithms were rarely available. Consequently, the current body of evidence demonstrates substantial technological promise but comparatively limited implementation readiness.

An additional limitation consistently identified throughout the literature concerns dataset origin. A considerable proportion of AI algorithms currently proposed for forensic applications were originally developed using clinical radiology or surgical pathology material before being adapted to postmortem investigation [17,18,19,20,24,25,26,27]. This distinction is particularly important because postmortem tissues differ fundamentally from living patients. Autolysis, decomposition, altered tissue attenuation, postmortem gas formation, blood redistribution, fixation variability, environmental exposure, and prolonged postmortem intervals generate biological conditions that are not adequately represented within conventional clinical datasets [21,22,23,31,32,46]. Consequently, excellent diagnostic performance reported in clinical environments cannot be assumed to translate directly into forensic practice without dedicated postmortem validation.

Beyond methodological limitations, the reviewed literature consistently emphasizes that forensic diagnosis differs fundamentally from most clinical applications of artificial intelligence. Clinical AI systems generally address relatively well-defined diagnostic questions, whereas medicolegal investigation requires integration of heterogeneous sources of evidence, including gross pathology, histopathology, toxicology, microbiology, molecular diagnostics, radiology, scene investigation, police reports, witness testimony, medical history, and legal standards [31,32,42,43,44,45]. Cause-of-death determination and medicolegal interpretation therefore extend beyond image recognition or pattern classification, requiring contextual reasoning, multidisciplinary synthesis, and professional accountability that currently remain outside the capabilities of existing AI systems.

The growing literature on explainable artificial intelligence further reinforces this interpretation. Recent methodological and regulatory publications increasingly emphasize that high-stakes medical applications require transparency, reproducibility, interpretability, and continuous human oversight [40,41,42,43,44,45]. These requirements are particularly stringent in forensic medicine because expert conclusions may directly influence criminal investigations, judicial proceedings, civil litigation, insurance disputes, and public health policy. Consequently, algorithmic performance should not be evaluated solely according to conventional diagnostic metrics such as accuracy or area under the receiver operating characteristic curve but also according to explainability, robustness, traceability, bias mitigation, cybersecurity, and evidentiary reliability [40,41,42,43,44,45].

Taken together, the available evidence supports a more balanced interpretation of AI than is often presented in the broader technological literature. Rather than functioning as an autonomous diagnostic system, artificial intelligence currently performs best as a decision-support technology capable of augmenting expert forensic practice through computational analysis, quantitative image interpretation, workflow optimization, multimodal data integration, and reduction in observer variability. This concept of augmented forensic intelligence is fully consistent with the conclusions of the most recent systematic reviews, which uniformly emphasize that AI should complement rather than replace forensic expertise [31,32,46].

Importantly, this interpretation aligns closely with the broader transformation discussed in the previous section. Within a digitally integrated model of death investigation, artificial intelligence should not be regarded as an independent technology competing with conventional autopsy or forensic expertise. Instead, it constitutes one computational component of an integrated investigative ecosystem in which radiological imaging, pathology, molecular diagnostics, toxicology, digital documentation, and human expert interpretation interact continuously throughout the medicolegal investigation. The current literature therefore supports the view that the future of forensic AI lies not in autonomous decision-making but in strengthening the diagnostic capability, reproducibility, efficiency, and analytical capacity of the forensic pathologist while preserving human responsibility for final medicolegal interpretation.

### 4.4. Precision Forensic Pathology: From Morphological Diagnosis to Multimodal Forensic Interpretation

The concept of precision medicine has profoundly influenced contemporary clinical practice by promoting individualized diagnosis and treatment through the integration of imaging, molecular biology, genomics, digital pathology, computational analysis, and clinical information. Rather than relying on isolated diagnostic tests, precision medicine seeks to combine complementary biological data into a comprehensive and patient-specific diagnostic framework [24,25,26,27]. Recent advances in computational pathology, artificial intelligence, and multimodal biomedical data integration have further strengthened this paradigm, demonstrating that diagnostic accuracy may be improved when heterogeneous sources of information are interpreted collectively rather than independently [17,18,19,20,21,22,23,24,25,26,27].

Digital pathology has been one of the principal drivers of this transformation. Landmark investigations by Campanella et al., Bera et al., and van der Laak et al. demonstrated that whole-slide imaging combined with computational pathology and deep-learning algorithms can achieve diagnostic performance approaching expert pathologists for selected clinical applications while simultaneously enabling quantitative morphometry, biomarker prediction, image segmentation, and remote consultation [17,18,19,20]. Although these developments were initially established within clinical pathology, subsequent forensic validation studies demonstrated that digital pathology can also be successfully applied to medicolegal practice, provided that postmortem-specific challenges—including autolysis, decomposition, fixation variability, and heterogeneous tissue preservation—are adequately addressed [21,22,23]. Recent systematic reviews therefore conclude that digital pathology has moved beyond proof of concept and now represents one of the most promising components of future forensic diagnostic workflows, although widespread implementation still requires standardized validation protocols and rigorous quality assurance [22,23].

A similar evolution has occurred in molecular diagnostics. Molecular autopsy has become an established complementary investigation in selected cases of sudden unexplained death, particularly those associated with inherited arrhythmogenic disorders and cardiomyopathies [33,34]. More recently, transcriptomics, epigenetics, proteomics, metabolomics, microbiome analysis, and postmortem microRNA profiling have expanded the diagnostic spectrum of forensic medicine by providing biological information that cannot be obtained through conventional anatomical examination alone [35,36,37,38]. Although these omics-based technologies remain at relatively early stages of forensic implementation, the available evidence consistently indicates that they complement rather than replace traditional pathology, extending medicolegal investigation into molecular mechanisms that remain morphologically silent.

Postmortem imaging has evolved in parallel. PMCT, PMMRI, PMCTA, and three-dimensional reconstruction now provide comprehensive radiological information before invasive examination, improving trauma documentation, vascular assessment, ballistic reconstruction, and minimally invasive investigation [10,11,12,13,14,15,16]. When interpreted together with histopathology, toxicology, microbiology, and molecular findings, these imaging modalities contribute information that extends beyond the capabilities of any individual technique. Rather than functioning as isolated diagnostic tools, they increasingly operate as complementary components of integrated forensic investigation.

Artificial intelligence represents a further layer within this multidimensional framework. As discussed previously, current evidence supports AI primarily as a computational technology capable of assisting quantitative image analysis, computational pathology, multimodal integration, and workflow optimization rather than autonomous medicolegal interpretation [24,25,26,27,28,29,30,31,32,46]. Importantly, the greatest potential of AI may not lie in improving the performance of individual technologies but in facilitating integration among heterogeneous datasets generated throughout forensic investigation. Emerging multimodal computational models increasingly seek to combine radiological images, histopathological findings, molecular analyses, toxicological results, scene documentation, and clinical information within unified analytical platforms capable of supporting expert decision-making [24,25,26,27,31,32].

Collectively, these technological developments indicate that forensic diagnosis is progressively moving beyond an exclusively morphology-based discipline. Historically, medicolegal interpretation depended primarily on macroscopic examination, histopathology, toxicology, microbiology, and circumstantial investigation. Contemporary forensic practice increasingly incorporates additional layers of biological and computational information that substantially expand diagnostic capability while preserving the central role of conventional pathological examination. Importantly, this transformation does not imply abandonment of morphological diagnosis; rather, it reflects the progressive integration of structural, molecular, radiological, toxicological, computational, and contextual evidence within a single interpretative framework.

Despite these advances, the literature also emphasizes that Precision Forensic Pathology has not yet been fully established as a standardized discipline. Current implementation remains heterogeneous across institutions, validation studies are uneven among technological domains, and internationally accepted operational frameworks have yet to be developed [22,23,31,32,42,43,44,45]. Furthermore, integration of multimodal forensic datasets continues to present important technical, ethical, regulatory, and organizational challenges, including interoperability, data governance, explainability of AI systems, digital chain of custody, and long-term validation of computational tools.

Nevertheless, when the available evidence is interpreted collectively, an important conceptual conclusion emerges. The defining characteristic of contemporary forensic pathology is no longer the isolated application of increasingly sophisticated technologies but their progressive convergence into integrated diagnostic workflows. In this context, Precision Forensic Pathology may be understood as the systematic integration of anatomical examination, postmortem imaging, digital pathology, molecular diagnostics, toxicology, computational analysis, and contextual medicolegal information to produce a more comprehensive, reproducible, and biologically informed interpretation of death. This concept does not replace conventional autopsy; instead, it extends its diagnostic capabilities through the coordinated use of complementary technologies, providing the conceptual bridge between digitally integrated death investigation and the paradigm shift discussed in the following section.

### 4.5. Does the Current Evidence Support a Genuine Paradigm Shift in Autopsy Pathology?

The concept of a scientific paradigm shift should be applied with considerable caution. As originally formulated by Thomas S. Kuhn in The Structure of Scientific Revolutions, scientific revolutions are not characterized simply by the introduction of new technologies or incremental methodological improvements. Rather, they occur when the conceptual framework through which a scientific community defines problems, generates evidence, validates knowledge, and conducts professional practice undergoes fundamental transformation [47]. Rather, they occur when the conceptual framework through which a scientific community defines problems, generates evidence, validates knowledge, and conducts professional practice undergoes fundamental transformation. Accordingly, the central question addressed by the present review is not whether artificial intelligence or digital technologies have improved forensic investigation, but whether the cumulative evidence indicates that autopsy pathology is experiencing a transformation of comparable conceptual significance.

The first characteristic supporting such an interpretation concerns the transformation of evidence acquisition. For most of its history, forensic investigation relied almost exclusively on direct anatomical examination supported by ancillary laboratory analyses. The literature reviewed demonstrates that this model has progressively expanded through the incorporation of PMCT, PMMRI, PMCTA, three-dimensional surface scanning, image-guided biopsy, and digital reconstruction, allowing forensic evidence to be acquired through multiple complementary modalities before conventional dissection begins [10,11,12,13,14,15,16]. Importantly, these technologies do not merely increase the quantity of available information; they fundamentally modify how anatomical evidence is generated, documented, and subsequently interpreted.

A second transformation involves the preservation and reproducibility of forensic evidence. Conventional autopsy inevitably alters anatomical structures during dissection, limiting subsequent independent reassessment. In contrast, postmortem imaging, whole-slide imaging, digital photography, three-dimensional reconstruction, and computational archiving generate permanent digital datasets that can be reviewed repeatedly without degradation of the original evidence [10,11,12,13,14,15,16,17,18,19,20,21,22,23]. These technologies substantially strengthen reproducibility, facilitate multidisciplinary consultation, improve quality assurance, and enhance judicial transparency. Consequently, forensic evidence increasingly evolves from a transient observation recorded in written reports into a permanent digital resource available for continuous scientific and medicolegal review.

A third characteristic involves the transformation of diagnostic interpretation. Historically, medicolegal diagnosis relied predominantly on the integration of gross pathology, histopathology, toxicology, microbiology, and circumstantial information interpreted by the forensic pathologist. Contemporary forensic investigation increasingly incorporates computational pathology, molecular autopsy, quantitative image analysis, artificial intelligence, and multimodal data integration as additional analytical layers [17,18,19,20,21,22,23,24,25,26,27,28,29,30,31,32,33,34,35,36,37,38]. Although none of these technologies currently replace expert interpretation, they substantially modify the diagnostic process by introducing computational and molecular information that extends beyond traditional morphological assessment. As discussed in the previous sections, recent systematic reviews consistently conclude that these technologies should function as complementary decision-support tools rather than autonomous diagnostic systems [31,32,46].

The reviewed evidence also demonstrates a profound transformation of investigative workflow. Conventional forensic investigation has traditionally followed a sequential structure in which anatomical examination, ancillary laboratory analyses, and final interpretation occurred as relatively independent stages. Increasingly, however, modern forensic practice operates through integrated workflows in which postmortem imaging guides tissue sampling, digital pathology supports computational analysis, molecular findings are interpreted alongside histopathology, and artificial intelligence assists multimodal synthesis of heterogeneous datasets [15,21,22,23,27,31,32]. This multidirectional interaction represents a substantial organizational departure from the traditional linear model of autopsy investigation.

Finally, the literature indicates that the professional role of the forensic pathologist is itself undergoing transformation. Contemporary forensic expertise increasingly requires competencies extending beyond conventional anatomical pathology to include forensic radiology, digital pathology, molecular diagnostics, computational pathology, artificial intelligence, data governance, and digital evidence management [21,22,23,24,25,26,27,40,41,42,43,44,45]. The forensic pathologist therefore functions not only as a specialist in postmortem anatomy but also as an integrator of radiological, molecular, computational, toxicological, and contextual information. Importantly, this evolution expands rather than diminishes the central scientific responsibility of the forensic pathologist, whose interpretative judgment remains indispensable for medicolegal decision-making.

When these observations are interpreted collectively, the available evidence supports a broader conceptual conclusion. The contemporary transformation of autopsy pathology cannot be explained solely by the introduction of isolated technological innovations. Instead, the literature consistently demonstrates simultaneous changes in evidence acquisition, evidence preservation, diagnostic interpretation, investigative workflow, and professional expertise. These interconnected transformations collectively modify the conceptual framework through which forensic investigation is conducted, interpreted, validated, and communicated.

Accordingly, when examined through the epistemological framework proposed by Kuhn [47], contemporary forensic pathology appears to satisfy multiple characteristics of a genuine scientific paradigm shift. Importantly, this conclusion should not be interpreted as evidence that conventional autopsy is becoming obsolete. On the contrary, every major technological domain reviewed continues to rely upon conventional autopsy as its biological reference standard for development, validation, and clinicopathological correlation. The paradigm shift identified in the present review therefore resides not in the replacement of conventional autopsy, but in the emergence of a hybrid, digitally integrated, and computationally augmented model of death investigation in which conventional pathology remains the indispensable scientific foundation while complementary technologies collectively redefine how medicolegal evidence is generated, integrated, interpreted, and communicated.

### 4.6. Conventional Autopsy as the Biological Reference Standard of Digitally Integrated Death Investigation

Scientific innovation requires reliable reference standards against which new technologies can be developed, validated, and critically evaluated. Throughout the history of modern medicine, conventional autopsy has fulfilled this role by providing the definitive anatomical and pathological correlation between disease processes and clinical manifestations. Numerous observational studies and systematic reviews continue to demonstrate that conventional autopsy remains the most reliable method for establishing the cause of death, identifying major diagnostic discrepancies, validating premortem diagnoses, and generating the pathological ground truth required for diagnostic quality assurance and biomedical research [2,3,4,5]. Consequently, the contemporary transformation of forensic pathology should not be interpreted as a progressive displacement of conventional autopsy but rather as an expansion of its diagnostic ecosystem.

This principle is particularly evident in postmortem imaging. PMCT, PMMRI, PMCTA, and related imaging technologies have undergone extensive validation over the past two decades; however, virtually all landmark validation studies have compared imaging findings directly with conventional autopsy [10,11,12,13,14,15,16]. The prospective investigation by Roberts et al. demonstrated that PMCT provides excellent diagnostic performance for skeletal trauma, intracranial hemorrhage, gas collections, and foreign-body localization while simultaneously confirming that many natural diseases remain dependent on conventional pathological examination [13]. Likewise, subsequent studies evaluating PMCTA consistently relied on autopsy findings as the reference standard for assessing vascular injuries, hemorrhagic lesions, and cardiovascular pathology [14,15,16]. These investigations illustrate that postmortem imaging derives its scientific credibility not from technological sophistication alone but from continuous comparison with conventional autopsy.

A similar relationship exists in digital pathology. Whole-slide imaging, computational pathology, and artificial intelligence-assisted histopathological analysis have demonstrated remarkable diagnostic potential in both clinical and forensic settings [17,18,19,20,21,22,23]. Nevertheless, validation studies consistently compare digital interpretation with conventional light microscopy performed on tissue obtained during standard autopsy procedures. Even recent forensic validation studies reporting excellent concordance between digital slides and conventional microscopy emphasize that successful implementation depends upon high-quality tissue sampling, standardized histological preparation, and expert pathological interpretation [21,22,23]. Thus, digital pathology extends the analytical capabilities of forensic medicine while remaining fundamentally anchored in conventional histopathological practice.

The same dependency is observed in molecular autopsy. Postmortem genetic testing has become an established adjunct in the investigation of sudden unexplained death associated with inherited arrhythmogenic disorders and cardiomyopathies [33,34]. However, molecular findings rarely possess sufficient diagnostic meaning when interpreted in isolation. Current international recommendations consistently advocate integrated clinicopathological correlation combining molecular results with gross examination, histopathology, toxicology, family history, and circumstantial information [33,34,35,36,37,38]. Accordingly, molecular autopsy should be regarded as a complementary extension of conventional autopsy rather than an alternative investigative strategy.

Artificial intelligence exhibits perhaps the clearest dependence on conventional pathology. Supervised machine-learning algorithms require accurately annotated reference datasets for training, validation, and performance assessment. In forensic medicine, these reference datasets are generated by experienced forensic pathologists through conventional autopsy, histopathological examination, and comprehensive medicolegal investigation [24,25,26,27,28,29,30,31,32,46]. Consequently, the biological “ground truth” upon which contemporary forensic AI depends is not created by computational systems themselves but by conventional pathological examination. Without reliable autopsy-derived reference standards, algorithm development, external validation, and clinical implementation would lack scientific credibility.

This dependence extends beyond technological validation to contemporary regulatory frameworks. International guidance issued by the World Health Organization, the European Union Artificial Intelligence Act, and the United States Food and Drug Administration consistently emphasizes that AI should be implemented under conditions of continuous human oversight, rigorous validation, transparency, explainability, and accountability [42,43,44,45]. Within forensic medicine, these principles acquire particular importance because medicolegal conclusions may directly influence criminal investigations, judicial decisions, insurance litigation, and public health policy. Human expert interpretation therefore remains an indispensable component of scientifically reliable and legally defensible forensic investigation.

Taken together, the reviewed evidence supports an important conceptual conclusion. Conventional autopsy should no longer be viewed simply as one diagnostic modality among many emerging technologies. Rather, it occupies a unique epistemological position as the biological reference standard upon which virtually every component of digitally integrated death investigation ultimately depends. Postmortem imaging is validated against autopsy findings; digital pathology depends upon autopsy-derived tissue; molecular autopsy requires clinicopathological correlation; and artificial intelligence relies upon expertly annotated autopsy datasets for algorithm development and validation. Thus, the central role of conventional autopsy has not diminished in the digital era. On the contrary, its importance has become even more fundamental because it provides the biological foundation that ensures the scientific validity, interpretative reliability, and medicolegal credibility of all currently available emerging technologies. The paradigm shift discussed in the previous section therefore does not replace conventional autopsy; it redefines its role as the indispensable scientific core of a hybrid, digitally integrated model of death investigation.

### 4.7. Quality Assurance, Validation, and Medico-Legal Admissibility: Toward a Forensic Validation Framework

The successful implementation of emerging technologies in forensic pathology depends on considerably more than technological innovation. In many areas of clinical medicine, new diagnostic tools are primarily evaluated according to analytical performance, diagnostic accuracy, and clinical effectiveness. In forensic medicine, however, technological performance alone is insufficient. Because medicolegal conclusions may directly influence criminal investigations, judicial proceedings, civil litigation, insurance claims, and public health decisions, every new technology must simultaneously satisfy scientific, technical, ethical, and legal standards of reliability [40,41,42,43,44,45]. Consequently, implementation of artificial intelligence and other digital technologies requires a validation process that extends well beyond conventional measures of diagnostic accuracy.

The literature reviewed consistently demonstrates that validation in forensic medicine should be understood as a multilevel process. Analytical validation establishes whether a technology performs its intended technical function under controlled conditions. Clinical validation determines whether that technology accurately detects or characterizes the biological phenomenon under investigation. Forensic validation, however, introduces an additional and distinct dimension by requiring demonstration that the technology remains reliable under authentic medicolegal conditions characterized by postmortem change, heterogeneous tissue preservation, decomposition, variable imaging protocols, complex mechanisms of death, and legally defensible interpretation [21,22,23,31,32,46]. This third level of validation remains comparatively underdeveloped across many emerging forensic technologies and represents one of the principal limitations identified throughout the current literature.

External validation constitutes another critical requirement. Recent systematic reviews consistently emphasize that many published artificial intelligence studies rely on retrospective, single-center datasets with limited demographic diversity, heterogeneous annotation strategies, and relatively small sample sizes [31,32,46]. Such studies frequently demonstrate excellent internal performance but provide comparatively little evidence regarding reproducibility across different forensic institutions, imaging protocols, geographic populations, or medicolegal systems. Robust multicenter validation, independent benchmarking datasets, standardized annotation protocols, and prospective implementation studies therefore remain essential prerequisites before AI systems can be incorporated into routine forensic practice.

Beyond diagnostic performance, explainability has emerged as a central requirement for responsible implementation of artificial intelligence. In forensic medicine, experts are expected not only to reach scientifically sound conclusions but also to explain the reasoning underlying those conclusions in a transparent, reproducible, and legally defensible manner. Black-box algorithms capable of generating highly accurate predictions without interpretable reasoning may therefore present important challenges in judicial environments, where expert testimony is subject to cross-examination and evidentiary scrutiny. Contemporary methodological frameworks consequently advocate explainable and interpretable AI systems capable of supporting, rather than obscuring, expert medicolegal reasoning [40,41,42,43,44,45].

Digital integrity represents an equally important component of forensic quality assurance. Unlike conventional laboratory diagnostics, digitally integrated forensic investigation increasingly depends upon complex information ecosystems incorporating postmortem imaging, whole-slide histopathology, molecular analyses, three-dimensional reconstructions, computational outputs, and AI-assisted interpretation. Maintaining the integrity of these digital datasets requires secure metadata management, comprehensive audit trails, version control, cybersecurity measures, authenticated digital storage, and robust chain-of-custody procedures capable of preserving evidentiary reliability throughout the entire lifecycle of digital forensic evidence [42,43,44,45]. Consequently, digital governance should be regarded as an integral component of forensic quality assurance rather than merely an information technology issue.

Quality assurance must therefore encompass the entire forensic workflow rather than isolated technological components. Pre-analytical factors—including body handling, imaging acquisition, tissue sampling, fixation, and specimen preservation—directly influence downstream computational analyses. Analytical performance depends on standardized laboratory procedures, validated algorithms, calibrated imaging systems, and reproducible computational pipelines. Finally, post-analytical interpretation requires expert pathological correlation, multidisciplinary review, transparent reporting, and continuous quality monitoring [21,22,23,31,32]. Failure at any stage may compromise the scientific validity of the entire investigative process regardless of the intrinsic performance of individual technologies.

When these observations are interpreted collectively, they support a broader conceptual model that may be described as a Forensic Validation Framework. Within this framework, implementation of emerging technologies requires the sequential integration of biological validation, analytical validation, forensic validation, external multicenter validation, explainability, digital governance, quality assurance, and medico-legal admissibility. Rather than functioning as independent requirements, these domains interact continuously to determine whether a technology is sufficiently robust for routine forensic application. This perspective shifts the focus of technological innovation away from isolated algorithmic performance toward comprehensive scientific reliability.

Accordingly, the future success of artificial intelligence and emerging technologies in forensic pathology will depend less on incremental improvements in computational performance than on the establishment of internationally accepted validation frameworks capable of ensuring biological credibility, methodological robustness, reproducibility, transparency, regulatory compliance, and judicial admissibility. Within the paradigm proposed in the present review, technological innovation should therefore be evaluated not simply by what new technologies can do, but by whether they satisfy the scientific and legal standards required for trustworthy medicolegal evidence. The development of such comprehensive validation frameworks may represent one of the most important priorities for the next generation of digitally integrated forensic pathology.

### 4.8. Regulatory Governance and Institutional Readiness for Digital Forensic Pathology

The rapid expansion of artificial intelligence and other digital technologies has been accompanied by equally rapid development of international regulatory initiatives intended to ensure their safe, transparent, and ethically responsible implementation. During the past decade, organizations including the World Health Organization (WHO), the United States Food and Drug Administration (FDA), and the European Union have progressively established regulatory principles addressing transparency, explainability, human oversight, lifecycle monitoring, algorithmic accountability, cybersecurity, and continuous quality assurance for AI-enabled healthcare technologies [42,43,44,45]. Collectively, these initiatives demonstrate an emerging international consensus that successful implementation depends not only on technological innovation but also on robust governance structures capable of ensuring scientific reliability and public trust.

Although these regulatory principles were developed primarily within clinical medicine, their relevance is arguably even greater in forensic pathology. Unlike most clinical diagnostic specialties, forensic medicine operates simultaneously within healthcare, criminal justice, civil litigation, insurance systems, occupational safety, and public health surveillance. Consequently, forensic conclusions frequently extend beyond patient care to influence judicial decisions, criminal investigations, compensation claims, and governmental policy. The evidentiary standards required for forensic technologies therefore encompass not only analytical performance but also legal admissibility, chain of custody, traceability, transparency, and long-term reproducibility [40,41,42,43,44,45]. These additional responsibilities distinguish forensic implementation from conventional clinical deployment and require governance models specifically adapted to the medicolegal environment.

The reviewed literature consistently suggests that technological readiness and institutional readiness should be regarded as distinct concepts. A technology may demonstrate excellent analytical performance under controlled research conditions while remaining unsuitable for routine forensic implementation if institutions lack standardized operating procedures, quality assurance systems, validated computational infrastructure, secure digital repositories, or personnel adequately trained to interpret digitally generated evidence [21,22,23,31,32,46]. Consequently, successful implementation depends not only upon algorithm development but also upon the organizational capacity of forensic institutions to incorporate new technologies into established medicolegal workflows.

Institutional readiness therefore requires substantially more than acquisition of advanced equipment or computational software. Contemporary forensic services increasingly require integrated governance structures encompassing standardized operating procedures, validated analytical protocols, quality management systems, digital chain-of-custody procedures, cybersecurity policies, metadata management, audit mechanisms, interoperability standards, long-term digital preservation, and continuous performance monitoring [42,43,44,45]. These organizational components are essential for ensuring that technological innovation translates into reproducible scientific practice rather than isolated technical capability.

An equally important challenge concerns professional education and workforce development. The reviewed evidence indicates that the future forensic pathologist will require competencies extending well beyond conventional anatomical pathology. Increasingly, forensic specialists must understand the principles of postmortem imaging, digital pathology, molecular diagnostics, computational pathology, artificial intelligence, data governance, cybersecurity, and multidisciplinary interpretation of multimodal forensic evidence [21,22,23,24,25,26,27,31,32]. This evolution has important implications for undergraduate medical education, pathology residency programs, forensic medicine training, and continuing professional development. Rather than replacing traditional pathological expertise, digital transformation expands the knowledge base required for competent medicolegal practice.

These observations suggest that digital transformation should be understood as an institutional rather than exclusively technological process. Successful implementation requires coordinated interaction among forensic pathologists, radiologists, molecular geneticists, computer scientists, engineers, information technology specialists, legal professionals, ethicists, and regulatory authorities. Accordingly, governance becomes a multidisciplinary institutional responsibility extending far beyond software regulation alone.

When interpreted collectively, the available evidence supports the proposal of an Institutional Readiness Framework for digitally integrated forensic pathology. Within this framework, successful implementation depends upon the coordinated development of technological infrastructure, standardized validation procedures, regulatory compliance, quality assurance systems, cybersecurity, professional education, multidisciplinary collaboration, and continuous institutional governance. These elements should not be regarded as independent requirements but as interdependent components of a mature digital forensic ecosystem capable of ensuring scientific credibility, operational sustainability, and judicial confidence.

Accordingly, the future of digitally integrated death investigation will depend not only on the continued evolution of artificial intelligence, molecular diagnostics, imaging technologies, or computational pathology, but also on the capacity of forensic institutions to develop governance structures capable of integrating these technologies safely, transparently, and sustainably into routine medicolegal practice. Institutional readiness may therefore represent one of the decisive determinants of whether the paradigm shift described in the present review will achieve widespread and responsible implementation.

### 4.9. Future Perspectives: The Next Frontier of Digitally Integrated Forensic Pathology

The evidence reviewed throughout this study indicates that forensic pathology is currently undergoing a period of accelerated technological convergence rather than isolated technological innovation. Nevertheless, the digital transformation described in the preceding sections should be regarded as an intermediate stage rather than the final destination of contemporary forensic medicine. The next phase of development will likely be characterized by deeper integration of existing technologies, increasing computational sophistication, and the establishment of internationally coordinated forensic ecosystems capable of generating, validating, and interpreting multimodal medicolegal evidence within unified digital environments.

One of the most promising directions involves the development of multimodal forensic intelligence. Current artificial intelligence systems generally analyze isolated datasets, such as postmortem CT images, histopathological slides, toxicological findings, or molecular analyses. Emerging computational models increasingly seek to integrate these heterogeneous data sources into unified decision-support systems capable of simultaneously processing radiological, pathological, molecular, toxicological, anthropological, scene-investigation, and contextual information [24,25,26,27,31,32]. Such multimodal approaches may substantially improve diagnostic consistency while preserving the central role of expert medicolegal interpretation.

Another rapidly evolving field concerns the emergence of foundation models trained on very large biomedical datasets. Unlike conventional task-specific algorithms, foundation models possess the potential to transfer learned representations across multiple diagnostic tasks and therefore may facilitate broader implementation of computational tools in forensic medicine [27]. Similarly, self-supervised learning may overcome one of the principal limitations identified throughout the present review by allowing algorithms to learn from large collections of unlabeled postmortem images and histopathological material, thereby reducing dependence on manually annotated datasets. However, current evidence indicates that these approaches remain largely experimental in forensic pathology and require extensive validation using authentic postmortem material before routine implementation can be considered.

The future of forensic pathology will also depend increasingly on international collaboration. Many of the limitations identified throughout this review—including limited external validation, relatively small datasets, heterogeneous annotation strategies, and institutional variability—cannot realistically be overcome by individual forensic centers acting independently [31,32,46]. Future progress will therefore require multicenter collaborative networks capable of generating standardized forensic repositories, harmonized imaging protocols, interoperable digital pathology platforms, curated molecular databases, shared benchmarking datasets, and internationally accepted validation standards. Such collaborative infrastructures may prove as important as future technological advances themselves.

Perhaps the greatest transformation, however, will occur at the level of scientific methodology rather than technology. As the digital forensic ecosystems mature, future research will increasingly shift its emphasis from demonstrating algorithmic feasibility toward evaluating clinical effectiveness, biological validity, operational impact, cost-effectiveness, reproducibility, explainability, and real-world implementation. Prospective multicenter studies, standardized reporting guidelines, continuous post-deployment monitoring, and transparent validation pipelines will therefore become essential components of next-generation forensic research [40,41,42,43,44,45]. In this context, scientific rigor may become a more important determinant of successful implementation than incremental improvements in computational performance.

These developments also have important implications for the education of future forensic specialists. Training programs will progressively require competencies extending beyond traditional pathology to include forensic imaging, computational pathology, molecular diagnostics, artificial intelligence, bioinformatics, data science, digital governance, and interdisciplinary interpretation of multimodal forensic evidence. Rather than replacing classical pathological expertise, these additional competencies will expand the scientific role of the forensic pathologist within increasingly complex investigative environments.

Ultimately, the future described by the available literature is not one in which artificial intelligence replaces the forensic pathologist or conventional autopsy. Instead, it is one in which progressively integrated digital ecosystems enhance the acquisition, interpretation, validation, and communication of medicolegal evidence while preserving human expertise as the central element of scientific judgment. The greatest advances of the coming decade are therefore likely to result not from any single emerging technology but from the successful integration of validated technologies within scientifically robust, institutionally mature, and internationally harmonized systems of digitally integrated death investigation.

### 4.10. Practical Implications for Forensic Practice and Health Systems

The evidence synthesized throughout this review has important practical implications for forensic institutions, healthcare systems, academic centers, regulatory authorities, and policymakers responsible for modernizing medicolegal services. The technological transformation currently occurring in forensic pathology should not be interpreted as a simple process of equipment acquisition or software implementation. Rather, it represents a comprehensive reorganization of forensic workflows requiring coordinated advances in scientific validation, institutional governance, professional education, and multidisciplinary collaboration.

The first implication concerns the implementation strategy adopted by forensic institutions. The reviewed evidence consistently indicates that emerging technologies should be incorporated progressively according to their level of scientific maturity rather than simultaneously or indiscriminately. Technologies supported by robust validation—particularly PMCT, PMCTA, and selected applications of molecular autopsy—are currently suitable for broader implementation in appropriately equipped centers [10,11,12,13,14,15,16,33,34]. Conversely, artificial intelligence, computational pathology, advanced omics technologies, and robotic-assisted postmortem systems should presently be introduced as complementary decision-support resources within carefully validated workflows while continuing to undergo prospective evaluation [21,22,23,31,32,46].

A second implication concerns the development of standardized operational protocols. The transition toward digitally integrated death investigation requires comprehensive standard operating procedures governing image acquisition, tissue sampling, digital pathology workflows, molecular analyses, algorithm validation, quality assurance, metadata management, cybersecurity, digital chain of custody, and multidisciplinary reporting [21,22,23,40,41,42,43,44,45]. Standardization is essential not only for scientific reproducibility but also for ensuring that digitally generated forensic evidence remains legally robust and operationally consistent across institutions.

Professional education represents another strategic priority. The reviewed literature indicates that future forensic specialists will increasingly require competencies extending beyond traditional anatomical pathology. Contemporary practice demands familiarity with forensic imaging, molecular diagnostics, computational pathology, artificial intelligence, bioinformatics, digital governance, and multidisciplinary interpretation of multimodal forensic evidence [21,22,23,24,25,26,27,31,32]. Consequently, undergraduate medical curricula, pathology residency programs, forensic medicine training, and continuing professional development should progressively incorporate these emerging disciplines while preserving rigorous training in conventional autopsy and histopathology, which remain the scientific foundation of medicolegal investigation.

The modernization of forensic services also requires stronger interdisciplinary collaboration. Digitally integrated forensic investigation increasingly depends upon coordinated interaction among forensic pathologists, radiologists, molecular biologists, toxicologists, computer scientists, biomedical engineers, information technology specialists, legal professionals, ethicists, and public health authorities. Such collaboration should become an integral component of routine forensic practice rather than an exceptional research activity. Multidisciplinary case review may improve diagnostic consistency, facilitate implementation of emerging technologies, and strengthen the scientific quality of medicolegal conclusions.

From a health systems perspective, digital transformation also offers opportunities extending beyond individual forensic investigations. Permanent digital archives, standardized imaging repositories, computational pathology platforms, and interoperable molecular databases may substantially enhance quality assurance, epidemiological surveillance, disaster victim identification, medical education, forensic research, and international scientific collaboration. These infrastructures may also facilitate secondary review, expert consultation, and long-term preservation of forensic evidence while improving institutional transparency and public confidence.

Importantly, successful implementation should remain guided by scientific evidence rather than technological enthusiasm. Throughout the present review, the strongest and most consistent conclusion is that technological innovation alone does not improve forensic practice unless accompanied by rigorous biological validation, methodological standardization, institutional governance, continuous quality assurance, and professional oversight. Emerging technologies should therefore be adopted according to demonstrated forensic value rather than novelty, ensuring that scientific reliability remains the primary determinant of implementation decisions.

Ultimately, the practical future of forensic pathology is unlikely to be defined by the replacement of conventional autopsy with artificial intelligence or any other individual technology. Instead, the reviewed evidence supports the progressive consolidation of digitally integrated death investigation as a mature forensic model in which conventional autopsy, postmortem imaging, digital pathology, molecular diagnostics, computational analysis, and artificial intelligence function synergistically within validated institutional frameworks. Under this model, technological innovation enhances diagnostic capability, reproducibility, efficiency, and transparency while preserving the central scientific role of the forensic pathologist and the biological authority of conventional autopsy. The principal challenge for the coming decade will therefore be not the development of additional technologies, but their responsible, evidence-based, and institutionally sustainable integration into routine medicolegal practice.

## 5. Strengths and Limitations

The present review has several important strengths. First, unlike previous reviews that have generally examined individual technologies in isolation, it provides an integrated critical synthesis of postmortem imaging, digital pathology, computational pathology, molecular autopsy, robotics, artificial intelligence, emerging omics technologies, and contemporary regulatory frameworks within a unified conceptual perspective of digitally integrated death investigation. By examining these domains collectively, the review moves beyond a descriptive overview and proposes an evidence-based interpretation of how these technologies interact to reshape contemporary forensic pathology.

Second, the review emphasizes the quality of evidence rather than technological novelty. Throughout the manuscript, individual technologies were critically appraised according to their methodological robustness, forensic applicability, validation status, implementation readiness, and regulatory implications. Particular attention was devoted to distinguishing mature technologies already incorporated into forensic practice from emerging approaches that remain supported primarily by proof-of-concept studies or early validation cohorts.

Third, this review integrates scientific evidence with broader methodological, ethical, epistemological, and institutional perspectives. The discussion extends beyond technological performance to address issues of explainability, biological validation, digital governance, quality assurance, institutional readiness, and medico-legal admissibility. In doing so, it proposes a series of complementary conceptual frameworks—including Digitally Integrated Death Investigation, Augmented Forensic Intelligence, Precision Forensic Pathology, Forensic Validation Framework, and Institutional Readiness Framework—that synthesize current evidence into a coherent interpretation of the ongoing transformation of forensic pathology.

This review also has important limitations. First, it was intentionally designed as a structured narrative review informed by a systematic literature search rather than as a formal systematic review or meta-analysis. Because the available evidence encompasses highly heterogeneous technologies, study designs, outcome measures, and forensic applications, quantitative pooling of results was neither feasible nor methodologically appropriate.

Second, the maturity of the available evidence differs substantially across technological domains. While postmortem imaging and molecular autopsy are supported by relatively robust validation studies, much of the literature concerning artificial intelligence, computational pathology, robotics, and emerging omics technologies remains based on retrospective analyses, technical feasibility studies, or proof-of-concept investigations. Consequently, the strength of evidence varies considerably among the technologies reviewed.

Perhaps the most important limitation, however, reflects the nature of the field itself. Artificial intelligence, digital pathology, computational medicine, and molecular technologies are evolving at an exceptional pace, with new algorithms, validation studies, regulatory initiatives, and implementation strategies emerging continuously. As a result, any synthesis of the current literature inevitably represents a snapshot of a rapidly changing scientific landscape. Future technological developments may alter the relative maturity of individual technologies, refine current implementation strategies, or modify the balance between experimental innovation and routine forensic practice. For this reason, the future perspectives discussed in this review should not be interpreted as deterministic predictions but rather as evidence-informed interpretations of the trajectories that are currently emerging from the available literature.

Nevertheless, the principal conceptual conclusions of this review are unlikely to be substantially affected by future technological advances. Although individual technologies will continue to evolve, the broader trends identified—including increasing multimodal integration, progressive digitalization of forensic workflows, growing importance of scientific validation and governance, and the continuing central role of conventional autopsy as the biological reference standard—are consistently supported across multiple technological domains and are therefore expected to remain relevant despite ongoing scientific and technological evolution.

## 6. Conclusions

Autopsy pathology is undergoing one of the most significant transformations since the establishment of modern clinicopathological investigation. The evidence reviewed throughout this study demonstrates that postmortem imaging, digital pathology, molecular diagnostics, computational pathology, artificial intelligence, robotics, and emerging omics technologies are progressively reshaping the acquisition, preservation, interpretation, validation, and communication of medicolegal evidence. Importantly, however, these developments should not be interpreted as isolated technological innovations but as interconnected components of an increasingly integrated forensic ecosystem.

The literature consistently demonstrates that these technologies differ substantially in scientific maturity. Postmortem imaging has achieved broad forensic applicability and currently represents the most mature component of digitally integrated death investigation. Molecular autopsy has become an established complementary investigation in selected diagnostic scenarios, whereas digital pathology is entering progressive forensic implementation. Artificial intelligence has demonstrated considerable potential across multiple analytical tasks but remains largely supported by retrospective investigations, limited multicenter validation, and proof-of-concept studies. Emerging omics technologies and robotic-assisted postmortem systems continue to show promising perspectives while remaining at comparatively early stages of forensic development.

A central conclusion of the present review is that contemporary forensic pathology should no longer be understood as the isolated application of increasingly sophisticated technologies. Instead, the available evidence supports the emergence of digitally integrated death investigation, in which conventional autopsy, imaging, digital pathology, molecular diagnostics, toxicology, computational analysis, and artificial intelligence function synergistically within a unified diagnostic framework. This multimodal integration represents the defining characteristic of the ongoing transformation of forensic practice.

Equally important, the reviewed evidence consistently indicates that conventional autopsy remains the indispensable biological reference standard upon which virtually all emerging technologies continue to depend. Imaging modalities are validated against autopsy findings; digital pathology depends upon autopsy-derived tissue; molecular autopsy requires clinicopathological correlation; and artificial intelligence relies on expertly annotated pathological datasets generated through conventional forensic investigation. The current technological revolution therefore strengthens rather than diminishes the scientific centrality of conventional autopsy.

Beyond synthesizing the available evidence, this review proposes a series of complementary conceptual frameworks that together provide an integrated interpretation of the ongoing transformation of forensic pathology. The concepts of Digitally Integrated Death Investigation, Augmented Forensic Intelligence, Precision Forensic Pathology, Forensic Validation Framework, and Institutional Readiness Framework organize the available literature into a coherent conceptual model that links technological innovation with biological validation, scientific rigor, institutional governance, and responsible implementation. Rather than representing independent proposals, these frameworks describe complementary dimensions of the same scientific transformation.

When interpreted collectively, the available evidence supports the conclusion that contemporary autopsy pathology is undergoing a genuine scientific paradigm shift, understood not as the replacement of conventional autopsy by artificial intelligence or any other emerging technology, but as a fundamental transformation in the conceptual framework through which medicolegal evidence is generated, integrated, interpreted, validated, and communicated. Viewed through this perspective, the digital transformation of forensic pathology reflects an evolution of scientific methodology rather than a substitution of its biological foundations.

The future of forensic pathology will therefore depend less on the development of individual technologies than on their successful integration within scientifically validated, institutionally mature, ethically governed, and legally robust forensic systems. Future priorities should include prospective multicenter validation studies, standardized forensic datasets, explainable artificial intelligence, interoperable digital infrastructures, internationally harmonized quality assurance procedures, and governance models capable of ensuring transparency, reproducibility, and judicial reliability.

Ultimately, the future of autopsy pathology lies neither in preserving traditional practice unchanged nor in replacing it with autonomous digital technologies. Rather, it lies in the progressive consolidation of a hybrid, digitally integrated, and computationally augmented model of death investigation, in which conventional autopsy remains the biological foundation while emerging technologies collectively expand the diagnostic capability, reproducibility, scientific robustness, and societal value of forensic medicine. It is this transformation—not the isolated adoption of artificial intelligence—that constitutes the true paradigm shift in contemporary autopsy pathology.

## Figures and Tables

**Table 1:** Representative landmark studies included in the present review according to technological domain, study design, principal forensic application, and evidence maturity.

Reference	Technology	Study Design	Principal Application	Validation	Maturity
Thali et al.	PMCT	Validation	Trauma	Prospective	Established
Roberts et al.	PMCT	Validation	Adult autopsy	Prospective	Established
Grabherr et al.	PMCTA	Review	Vascular imaging	Multicenter	Established
Campanella et al.	Digital pathology	Deep learning	Histopathology	External	Advanced
Pigaiani et al.	Forensic WSI	Validation	Histopathology	Multicenter	Advanced
Garland et al.	AI	Feasibility	Head injury	Internal	Early
Zirn et al.	AI	Validation	Cerebral hemorrhage	Internal	Early
Zeng et al.	AI	Validation	Hypothermia	Internal	Early
Semsarian et al.	Molecular autopsy	Review	Sudden death	Clinical	Advanced
Bagnall et al.	Molecular autopsy	Prospective	Cardiac death	Prospective	Advanced
Ebert et al.	Robotics	Feasibility	Virtobot	Pilot	Proof of concept

**Table 2:** Technology maturity assessment of emerging technologies in contemporary autopsy pathology.

Technology	Current Maturity	Principal Strengths	Main Limitations	Current Forensic Applicability
Conventional autopsy	Reference standard	Comprehensive diagnosis	Invasive	Universal
PMCT	Established	Trauma, fractures	Microscopic disease	Routine
PMCTA	Established	Vascular lesions	Specialized infrastructure	Routine in specialized centers
Molecular autopsy	Advanced validation	Sudden cardiac death	Limited indications	Selected cases
Whole-slide imaging	Advanced validation	Remote consultation	Standardization	Increasing
Computational pathology	Early validation	Quantitative analysis	External validation	Emerging
AI-assisted PMCT	Early validation	Automated detection	Limited datasets	Emerging
AI-assisted histopathology	Early validation	Image analysis	Limited validation	Emerging
Robotics	Proof of concept	Standardization	Limited evidence	Experimental
Multi-omics	Experimental	Precision diagnosis	Standardization	Investigational

## Data Availability

No new datasets were generated or analyzed during this study. All information discussed is derived from published literature cited in the reference list.

## Supplementary Materials

The following supporting information can be downloaded at: https://www.mdpi.com/article/10.3390/diagnostics16152405/s1, Supplementary Materials File S1: PRISMA 2020 Checklist.

## Abbreviations

AI	Artificial Intelligence
CNN	Convolutional Neural Network
CT	Computed Tomography
DL	Deep Learning
LLM	Large Language Model
MRI	Magnetic Resonance Imaging
PMCT	Postmortem Computed Tomography
PMMRI	Postmortem Magnetic Resonance Imaging
WSI	Whole-Slide Imaging
