# Supplementary material for: Autopsy Pathology’s Paradigm Shift: Artificial Intelligence and Emerging Technologies in the Era of Digitally Integrated Death Investigation
Source: Diagnostics (Basel). 2026 Jul 30;16(15):2405. doi: 10.3390/diagnostics16152405 (PMC13465568; doi:10.3390/diagnostics16152405)
Supplement: Supplementary file 1 [file diagnostics-16-02405-s001.zip › diagnostics-4422543-supplementary.pdf]

# Supplementary File S1. PRISMA 2020 Checklist

Manuscript ID: diagnostics-4419468
Autopsy Pathology's Paradigm Shift: Artificial Intelligence and Emerging Technologies in the Era of Digitally Integrated Death Investigation
Authors: Ivan Dieb Miziara; Carmen Silvia Molleis Galego Miziara

**Note.** This checklist is completed for a structured narrative review with a systematic literature search and qualitative synthesis. Items related to meta-analysis, quantitative effect estimates, certainty assessment, or risk-of-bias synthesis are marked as not applicable when they do not correspond to the manuscript design.

| Domain | Item | Checklist item | Location in manuscript | Author response / notes |
|---|---|---|---|---|
| Title | 1 | Identify the report as a review. | Title page | The manuscript is identified as a Review and describes a structured narrative review with systematic literature search. |
| Abstract | 2 | Provide a structured summary including background, objectives, data sources, eligibility, synthesis methods, results, limitations, and conclusions. | Abstract | Structured abstract includes background, objective, methods, results, conclusions. Add explicit note if desired: qualitative synthesis; no meta-analysis. |
| Introduction | 3 | Rationale for the review. | Introduction | The introduction explains decline of conventional autopsy and the rise in AI, postmortem imaging, digital pathology, molecular autopsy, robotics, and computational medicine. |
| Introduction | 4 | State the review objectives/questions. | End of Introduction; Abstract Objective | Objective stated: evaluate how AI and emerging technologies are transforming autopsy pathology and whether they support a paradigm shift. |
| Methods | 5 | Specify review registration or protocol information. | Materials and Methods / Study Design | Not registered. Suggested addition: no protocol was prospectively registered because this was a structured narrative review rather than a formal systematic review. |
| Methods | 6 | Specify eligibility criteria. | Materials and Methods / Eligibility Criteria | Eligibility criteria are provided, including technology domains, study types, forensic relevance, and exclusion of unrelated or insufficiently described records. |
| Methods | 7 | Describe information sources. | Materials and Methods / Search Strategy | PubMed/MEDLINE, Scopus, Web of Science, Embase, and reference-list screening are reported. |
| Methods | 8 | Present search strategies. | Materials and Methods / Search Strategy | Representative search string is provided. For full PRISMA compliance, consider adding complete database-specific search strings as Supplementary File S3 if requested. |
| Methods | 9 | Describe the selection process. | Materials and Methods / Study Selection and Synthesis; Figure 1 | Titles/abstracts and full texts were screened for relevance; disagreements resolved by author consensus. |
| Methods | 10 | Describe data collection/extraction process. | Materials and Methods / Study Selection and Synthesis | Extracted information included year, technological domain, forensic application, study type, findings, limitations, and forensic relevance. |
| Methods | 11 | List and define data items. | Materials and Methods / Study Selection and Synthesis; Tables 1-3 | Data items are described narratively and summarized by technology domain. |
| Methods | 12 | Describe risk-of-bias assessment. | Limitations | Not applicable as a formal risk-of-bias assessment was not performed; review is narrative and conceptually synthetic. The limitation should remain explicit. |
| Methods | 13 | Specify effect measures. | Not applicable | No quantitative effect measure or pooled estimate was planned because no meta-analysis was performed. |
| Methods | 14 | Describe synthesis methods. | Materials and Methods / Study Selection and Synthesis | Qualitative narrative synthesis grouped into technological domains: imaging, AI, digital pathology, molecular autopsy, robotics, quality assurance, and future models. |
| Methods | 15 | Describe reporting-bias assessment. | Not applicable / Limitations | Not applicable. Publication bias toward positive technology findings is acknowledged as a limitation. |
| Methods | 16 | Describe certainty/confidence assessment. | Not applicable | No GRADE or equivalent certainty assessment was performed because the objective was conceptual synthesis, not pooled evidence grading. |
| Results | 17 | Report study selection results, preferably with a flow diagram. | Results / Overview; Figure 1 | Figure 1 is described as a PRISMA-informed flow diagram. Numeric consistency must be checked against the final figure before submission. |
| Results | 18 | Present characteristics of included studies. | Results / Overview; Table 1 | Representative landmark studies and technological domains are summarized in Table 1. |
| Results | 19 | Present risk-of-bias results. | Not applicable | No formal risk-of-bias table is included; not applicable to the stated structured narrative review design. |

| | | | | |
|---|---|---|---|---|
| Results | 20 | Present results for individual studies. | Results / Table 1 and narrative subsections | Individual landmark studies are referenced in domain-based synthesis rather than full extraction tables. |
| Results | 21 | Present synthesis results. | Results; Discussion | Qualitative synthesis is organized by technology domain and forensic applicability. |
| Results | 22 | Present reporting-bias results. | Not applicable | No statistical reporting-bias assessment was performed. |
| Results | 23 | Present certainty/confidence results. | Not applicable | No certainty grading performed. |
| Discussion | 24 | Provide a general interpretation of results. | Discussion Sections 4.1-4.10 | Discussion interprets the evidence as a hybrid, digitally integrated, computationally augmented paradigm shift rather than replacement of conventional autopsy. |
| Discussion | 25 | Discuss limitations of evidence and review process. | Limitations | Limitations include narrative design, heterogeneity, experimental status of AI applications, lack of external validation, publication bias, and rapid technology evolution. |
| Discussion | 26 | Discuss implications for practice, policy, and research. | Discussion / Practical Implications; Conclusions | Implications include validation, quality assurance, medico-legal admissibility, training, governance, and hybrid forensic workflows. |
| Other information | 27 | Report funding, competing interests, data availability, and other relevant disclosures. | Funding; Conflicts of Interest; Data Availability; AI-Assisted Content Disclosure | Funding, conflicts, data availability, and AI-assisted content disclosure are provided. |
